# Junior surgeons are quicker to master the single-port thoracoscopic lobectomy: comprehensive analysis of the learning curve and oncological outcomes

**DOI:** 10.1186/s12957-023-03017-6

**Published:** 2023-04-22

**Authors:** Mingqiang Liang, Peixun Wu, Chi Xu, Bin Zheng, Chun Chen

**Affiliations:** 1 Department of Thoracic Surgery, Fujian Medical University Union Hospital, #29 Xinquan Road, Fujian 350001 Fuzhou, China; 2 Key Laboratory of Cardio-Thoracic Surgery (Fujian Medical University), Fujian Province University, Fuzhou, 350001 Fujian China

**Keywords:** Thoracic surgeons, Single-port thoracoscopic lobectomy, Lung cancer, Learning curve, Oncological outcome

## Abstract

**Background:**

The learning curve of single-port thoracoscopic lobectomy (SPTL) in lung cancer has been widely studied. However, the efficiency of different experience levels of thoracic surgeons in mastering the learning curve is unknown. Hence, we discuss this issue in depth by using several perioperative parameters and oncological outcomes.

**Methods:**

A total of 120 consecutive cases of SPTL performed by a senior (STS group) and junior (JTS group) thoracic surgeons were retrospectively analyzed. Operation time, estimated blood loss, and duration of postoperative hospital stay were recorded for cumulative summation (CUSUM) learning curve analysis, while the 5-year survival rate was used for oncological evaluation.

**Results:**

The CUSUM learning curve of the STS group was *y* = 0.000106x^3^ − 0.019x^2^ + 0.852x − 0.036, with a high R-value of 0.9517. When the number of cases exceeded 33, the slope changed from positive to negative. The CUSUM learning curve of the JTS group was *y* = 0.000266x^3^ − 0.04x^2^ + 1.429 × –0.335, with a high R-value of 0.9644. When the number of cases exceeded 25, the slope changed from positive to negative. The learning curve was divided into two phases (phases 1 and 2). The slope of the JTS group in phase 1 was greater than that of the STS group in phase 1 (*p* < 0.001). Meanwhile, comparisons of various parameters between both groups in phase 2 showed no statistically significant difference (*p* > 0.05). In addition, the 5-year survival rate was not significantly different between the two groups (*p* = 0.72).

**Conclusion:**

This is the first study to analyze the learning curve of thoracic surgeons with different experience levels in mastering SPTL. Moreover, it is also the first study to include multiple perioperative parameters and overall survival to study how quickly surgeons master the SPTL technique. The junior thoracic surgeon was found to have a shorter learning curve for SPTL.

## Introduction

Lung cancer remains the leading cause of cancer-related deaths worldwide, and its incidence and mortality have continued to increase in recent decades [1]. Lobectomy with systemic lymphadenectomy remains the standard curative treatment for lung carcinoma, despite alternative surgical approaches, including segmentectomy and pneumonectomy [2–4].

Traditional thoracoscopic lobectomy has gained worldwide acceptance [5–7]. However, in 2011, Gonzalez et al. reported their first experiences with single-port thoracoscopic lobectomy (SPTL) [8], and more complex procedures, such as segmentectomy [9], pneumonectomy [10], and sleeve lobectomy [11], have been described. To some extent, SPTL for lung cancer has been regarded as a suitable alternative to traditional thoracoscopic surgery owing to its advantages, including fewer traumas and pain, better cosmetic outcomes, quicker recovery, and shorter hospital stays [12–14].

Although SPTL has become increasingly popular, its disadvantages include a restricted range of motion and an uncomfortable position for the surgeon. Therefore, it is important to assess the learning curve for this method. However, in clinical practice, we found that thoracic surgeons with different experience levels have different efficiencies in mastering the learning curve of SPTL. Therefore, it is of great significance to discuss this issue in depth, which will help promote the extensive development of SPTL.

The learning curve of SPTL surgery has been studied previously. For example, Cheng et al. performed a retrospective study using the linear regression method to analyze the learning curve of single-port thoracoscopic segmentectomy [15], and Liu et al. used the cumulative summation (CUSUM) method to analyze the learning curve of SPTL, finding that 44 cases were required to overcome the learning curve [16]. Both studies used operation time as the only index and considered it sufficient for evaluating the learning curve, according to previous studies [17–19]. Therefore, we conducted a retrospective study to analyze the learning curve for SPTL using the multidimensional CUSUM method with three items, including operation time, estimated blood loss, and duration of postoperative hospital stay, which was uniquely suited for learning curve evaluation.

In addition, it has been reported that oncological outcomes are associated with the learning curve of minimally invasive surgery in several cancers [20–22]. In this study, to further verify the surgical competence, we compared the 5-year overall survival after the surgeon had overcame the learning curve.

## Materials and methods

A retrospective study was conducted to analyze 120 patients with clinical stage 1A lung cancer who underwent SPTL between May 2014 and June 2015 at the Department of Thoracic Surgery, Fujian Medical University Union Hospital. All patients received several routine examinations such as thoracic computed tomography (CT), cerebral magnetic resonance imaging (MRI), skeletal emission computed tomography (ECT), and cervical and abdominal color Doppler ultrasound (CDU). Positron emission tomography-CT (PET-CT) would be applicable when routine exam cannot rule out the underlying metastasis. Besides, pulmonary function test, electrocardiogram, and cardiac CDU were applied to assess cardiopulmonary function. The inclusion criteria were as follows: (1) non-small cell lung cancer diagnosis; (2) cancer diameter less than 3 cm and the absence of pleural dissemination, mediastinal lymph nodes, or distant organ metastasis; (3) pulmonary and cardiac function meeting the requirements for lobectomy; and (4) accompanying disorders stable after medical consent. The following groups of patients were excluded from the study: (1) patients with a previous history of chemotherapy, radiotherapy, or surgery, (2) patients with abnormal preoperative pulmonary and cardiac function and in whom thoracoscopic procedure was not tolerated, and (3) patients with pathology proven benign masses.

Sixty surgeries were performed by a senior thoracic surgeon (STS), and the remaining surgeries were performed by a junior thoracic surgeon (JTS). Both surgeons had plenty of experience in thoracic surgery and traditional thoracoscopic surgery, with approximately more than 10 years of experience in the case of the STS and less than 5 years of experience in the case of the JTS. The study was approved by the Ethics Committee of Fujian Medical University Union Hospital, and all procedures were performed in accordance with the Declaration of Helsinki.

### Surgery procedure

**Fig. 1:**
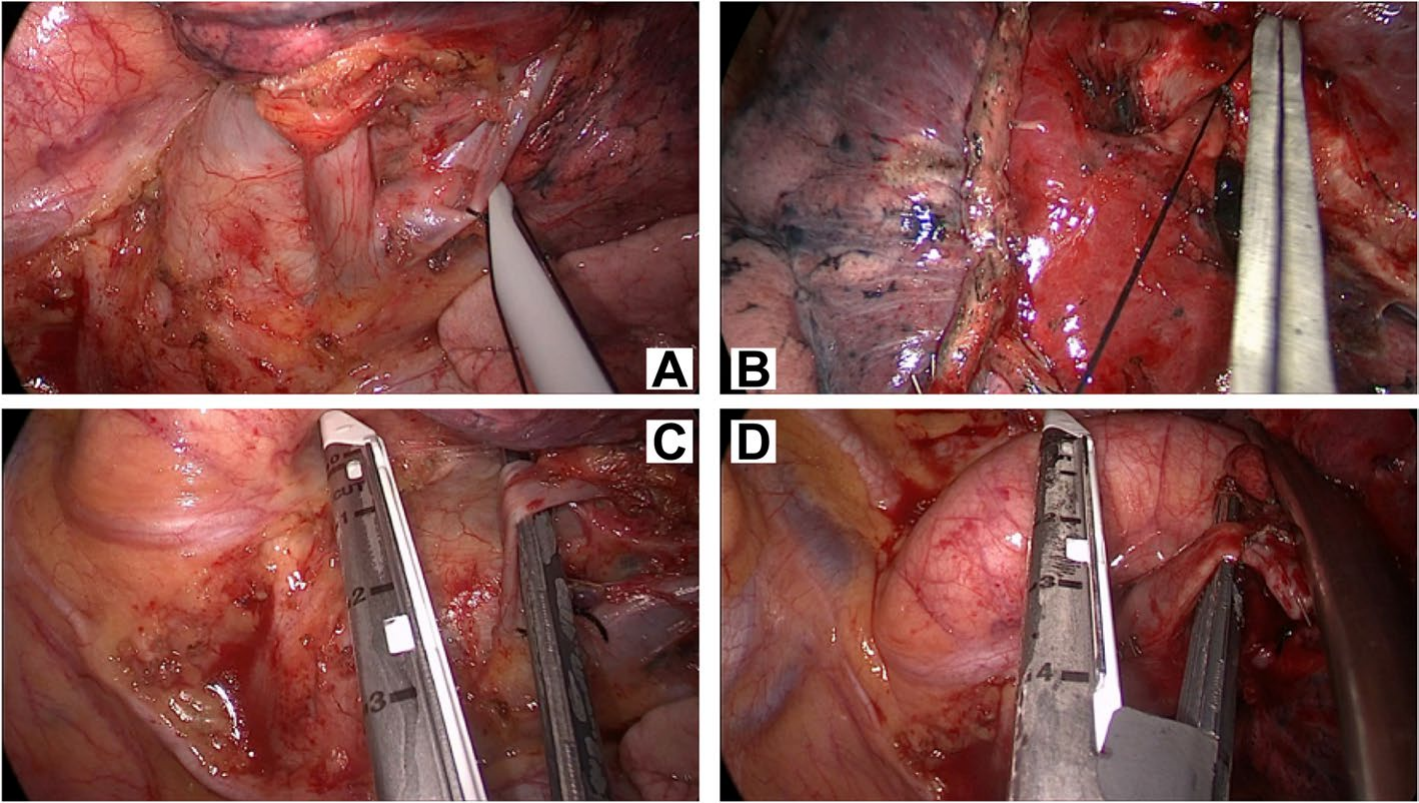
Typical images of the SPTL surgical procedure, especially the treatment of pulmonary vessels. Silk ligates the pulmonary vein (**A**) or artery (**B**), and the endostapler cuts the pulmonary vein (**C**) or artery (**D**). PTL, single-port thoracoscopic lobectomy

After anesthetization and double-lumen endotracheal intubation, the patient was placed in the lateral position. A 3.5-cm incision was made in the 4th or 5th inter-coastal space between the anterior axillary line. The primary procedures included lobectomy and systemic lymphadenectomy. Lymph nodes from the stations of 4, 5, 6, 7, 8, and 9 were dissected for left lung cancer and from stations of 2, 4, 7, 8, and 9 for right lung cancer. The pulmonary fissures were analyzed using the standard lung window settings (level: − 600 HU, width: 1600 HU); we divided the fissure in two categories: incomplete 1–40% and complete 60–100% [23]. The SPTL procedures were the same as those described previously [24, 25]. Some typical images regarding surgical procedure in SPTL are shown in Fig. 1.

### Data collection

All data, including clinical demographics, perioperative parameters, and 5-year survival rate, were recorded. Perioperative parameters included operation time, estimated blood loss, postoperative chest tube duration, duration of postoperative hospital stay, and postoperative complications. The operation time was considered as beginning when the incision was made until the time the incision was sutured. The indications for chest tube removal were as follows: (1) 24-h chest tube drained volume < 100 mL, (2) no bubbles during the forced active cough, and (3) postoperative chest X-ray show the left lung recruitment, and no effusion deposited. The discharge criteria in our study were that the patients recovered to normal mobility status, without obvious fever, after withdrawing the chest drainage. Follow-up was carried out from the moment when the patients were discharged until July 10, 2021, via outpatient records, telephones, letters, etc.

### CUSUM analysis

In our multidimensional CUSUM analysis, we designated operation time, estimated blood loss, and duration of postoperative hospital stay as the assessment indicators of surgical competence. These three assessment indicators were set as the quantized values a1, a2, and a3 for each case. The quantized value of the assessment indicator was defined as *a* = Xi–X0, where Xi is an individual attempt, with *Xi* = 1 for failure and *Xi* = 0 for success. Failure was defined as follows: (1) operation time 60 min above the average, (2) estimated blood loss 100 mL above the average, and (3) duration of postoperative hospital stay more than twice the average. X0 is the predetermined failure rate inherent to the procedure, and X0 was set to 10% for each indicator in our study. Therefore, the quantized value of surgical competence for each patient was defined as S = a1 + a2 + a3. After each case, scores were sequentially added and plotted graphically using the equation *CUSUM* = ∑Si.

The CUSUM values for each case were collected from the observations, and the corresponding scatter plots were obtained. The relationship between the CUSUM value (Y) and the number of cases (X) was determined using the curve-fitting method. The fitting curve was as close to each scatter point as possible. The relationship between *Y* and *X* was described by a one-variable cubic equation [26]. The coefficient R was used to determine the degree of dispersion of the CUSUM value and the fitted curve in the image. The larger the R-value, the higher the reliability of the equation function corresponding to the fitted curve and the more accurate the description of the learning process. A positive slope implied that the target had not been achieved, whereas a negative slope suggested that the target had been exceeded. The transition point of the slope from positive to negative indicated competence of the surgeon.

### Statistical analysis

Statistical analysis was performed using SPSS 22.0 for Windows (SPSS, Inc., Chicago, IL, USA) and Microsoft Office Excel 2016 (Microsoft, Inc., Redmond, TX, USA). Continuous variables were expressed as mean ± standard deviation (SD). For inter-group comparisons, the independent sample *t*-test or Mann–Whitney *U*-test was used in case of continuous variables after normality tests, and the chi-square test was used in case of categorical variables. The survival rate of both the groups was analyzed using Kaplan–Meier analysis, and the difference in the survival rate between the two groups was compared using the log-rank test. Statistical significance was set at *p* < 0.05 (two sided).

## Results

### Clinical characteristics

All the patients successfully underwent SPTL, without need for conversion. Age, sex, medical comorbidities (such as diabetes, hypertension, and cardiac diseases), histological type, tumor stage, and perioperative parameters are summarized in Table 1. No significant differences were observed between the groups.

**Table 1:** Parameters of both groups

Parameters	STS (*n* = 60)	JTS (*n* = 60)	*p*-value
Age (year)	56.8 ± 12.7	57.3 ± 12.7	0.83
Sex (female/male)	34/26	36/24	0.71
FEV1 (L)	1.8 ± 0.4	1.7 ± 0.3	0.63
Commonalities (yes/no)	7/53	10/50	0.43
Adhesion (yes/no)	5/55	4/56	0.73
Fissure (complete/incomplete)	46/14	39/21	0.16
Tumor location (RUL/RML/RLL/LUL/LLL)	19/4/15/14/8	16/3/13/16/12	0.27
Histology type (AC/SCC/others)	51/6/3	47/9/4	0.36
TNM stage (I/II/III)	38/12/10	44/11/5	0.19
Operation time (min)	215.3 ± 46.1	221.8 ± 59.0	0.49
Estimated blood loss (mL)	92.7 ± 57.0	92.6 ± 57.7	1.00
Number of lymph node harvested (*n*)	22.5 ± 7.9	22.1 ± 9.6	0.84
Chest tube duration (d)	5.7 ± 4.2	5.5 ± 3.5	0.43
Postoperative hospital stays (d)	8.7 ± 5.7	8.5 ± 5.2	0.83
Complications (*n*)	6	8	0.85
Air leaking	2	2	
Atrial fibrillation	1	1	
Chylothorax	1	1	
Pulmonary embolism	1	1	
Pulmonary infection	1	3	

*FEV1* forced expiratory volume in the first second, *RUL/RML/RLL/LUL/LLL* right upper lobe/right middle lobe/right lower lobe/left upper lobe/left lower lobe, *AC* adenocarcinoma, *SCC* squamous cell carcinoma

### CUSUM learning curves

#### STS group

**Fig. 2:**
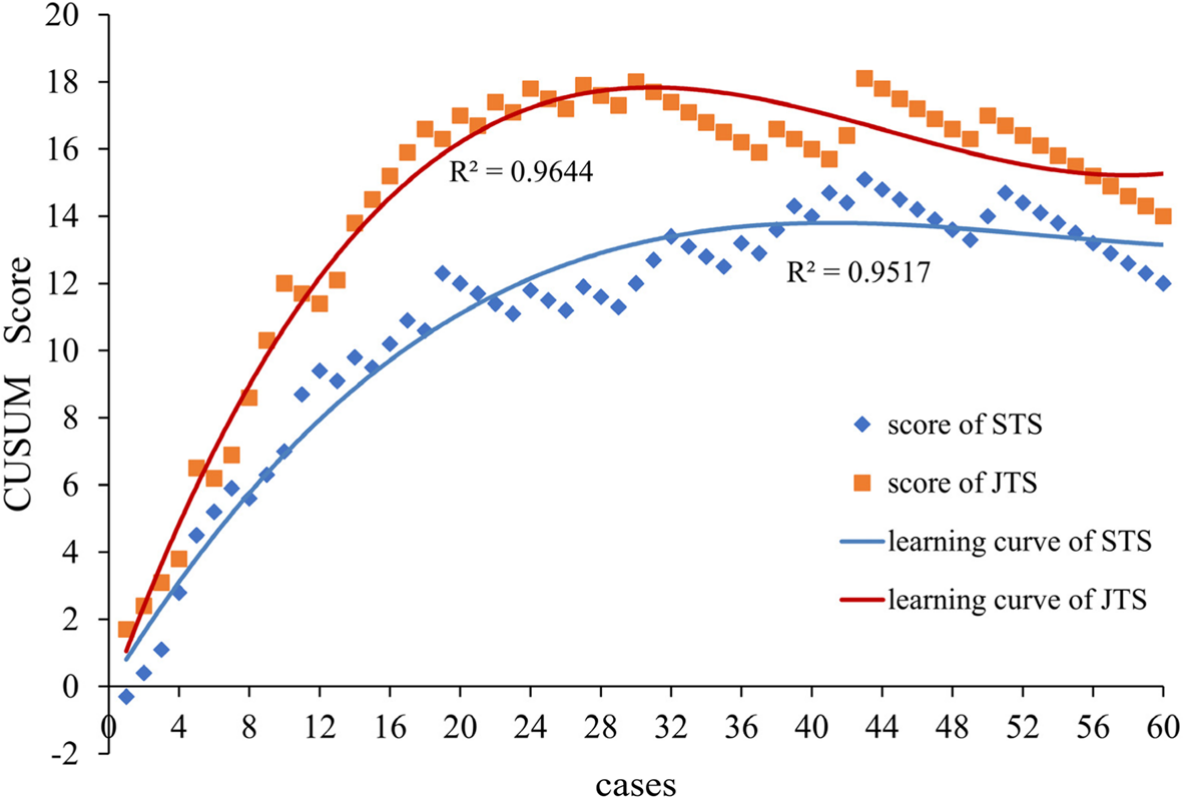
The graphs of the learning curve for both groups in single-port thoracoscopic lobectomy

The CUSUM learning curve of the STS group is shown in Fig. 2. The best fit for the curve was a third-order polynomial equation: *y* = 0.000106x^3^ − 0.019x^2^ + 0.852x − 0.036, which had a high R-value of 0.9517. The slope of the learning curve is shown in Table 2. For the STS, the slope changed from positive to negative when the number of cases exceeded 33. The learning curve was divided into two phases: phase 1, identified by the first 33 cases, and phase 2, identified by the remaining 27 cases.

**Table 2:** Cumulative summation score and slope score of the learning curve in single-port thoracoscopic lobectomy for senior thoracic surgeon

Cases	CUSUM	Slope	Cases	CUSUM	Slope	Cases	CUSUM	Slope
1	− 0.3	0.8	**21**	11.7	0.2	**41**	14.7	− 0.2
2	0.4	0.8	**22**	11.4	0.2	**42**	14.4	− 0.2
3	1.1	0.7	**23**	11.1	0.1	**43**	15.1	− 0.2
4	2.8	0.7	**24**	11.8	0.1	**44**	14.8	− 0.2
5	4.5	0.7	**25**	11.5	0.1	**45**	14.5	− 0.2
6	5.2	0.6	**26**	11.2	0.1	**46**	14.2	− 0.2
7	5.9	0.6	**27**	11.9	0.1	**47**	13.9	− 0.2
8	5.6	0.6	**28**	11.6	0.0	**48**	13.6	− 0.2
9	6.3	0.5	**29**	11.3	0.0	**49**	13.3	− 0.2
10	7	0.5	**30**	12	0.0	**50**	14	− 0.3
11	8.7	0.5	**31**	12.7	0.0	**51**	14.7	− 0.3
12	9.4	0.4	**32**	13.4	0.0	**52**	14.4	− 0.3
13	9.1	0.4	**33**	13.1	− 0.1*	**53**	14.1	− 0.3
14	9.8	0.4	**34**	12.8	− 0.1	**54**	13.8	− 0.3
15	9.5	0.4	**35**	12.5	− 0.1	**55**	13.5	− 0.3
16	10.2	0.3	**36**	13.2	− 0.1	**56**	13.2	− 0.3
17	10.9	0.3	**37**	12.9	− 0.1	**57**	12.9	− 0.3
18	10.6	0.3	**38**	13.6	− 0.1	**58**	12.6	− 0.3
19	12.3	0.2	**39**	14.3	− 0.1	**59**	12.3	− 0.3
20	12	0.2	**40**	14	− 0.2	**60**	12	− 0.3

*CUSUM* Cumulative summation score

*The slope changed from positive to negative for the first time

#### JTS group

The CUSUM learning curve of the JTS group is shown in Fig. 2. The best fit for the curve was a third-order polynomial equation: *y* = 0.000266x^3^ − 0.04x^2^ + 1.429x − 0.335, which had a high R-value of 0.9644. The slope of the learning curve is shown in Tables 2 and 3. In the JTS group, the slope changed from positive to negative when the number of cases exceeded 25. The learning curve was divided into two phases: phase 1, identified by the first 25 cases, and phase 2, identified by the subsequent 35 cases.

**Table 3:** Cumulative summation score and slope score of the learning curve in single-port thoracoscopic lobectomy for junior thoracic surgeon

Cases	CUSUM	Slope	Cases	CUSUM	Slope	Cases	CUSUM	Slope
1	1.7	1.3	**21**	16.7	0.1	**41**	15.7	− 0.5
2	2.4	1.3	**22**	17.4	0.1	**42**	16.4	− 0.5
3	3.1	1.2	**23**	17.1	0.0	**43**	18.1	− 0.5
4	3.8	1.1	**24**	17.8	0.0	**44**	17.8	− 0.5
5	6.5	1.0	**25**	17.5	− 0.1*	**45**	17.5	− 0.6
6	6.2	1.0	**26**	17.2	− 0.1	**46**	17.2	− 0.6
7	6.9	0.9	**27**	17.9	− 0.1	**47**	16.9	− 0.6
8	8.6	0.8	**28**	17.6	− 0.2	**48**	16.6	− 0.6
9	10.3	0.8	**29**	17.3	− 0.2	**49**	16.3	− 0.6
10	12	0.7	**30**	18	− 0.3	**50**	17	− 0.6
11	11.7	0.6	**31**	17.7	− 0.3	**51**	16.7	− 0.6
12	11.4	0.6	**32**	17.4	− 0.3	**52**	16.4	− 0.6
13	12.1	0.5	**33**	17.1	− 0.3	**53**	16.1	− 0.6
14	13.8	0.5	**34**	16.8	− 0.4	**54**	15.8	− 0.6
15	14.5	0.4	**35**	16.5	− 0.4	**55**	15.5	− 0.6
16	15.2	0.4	**36**	16.2	− 0.4	**56**	15.2	− 0.5
17	15.9	0.3	**37**	15.9	− 0.4	**57**	14.9	− 0.5
18	16.6	0.2	**38**	16.6	− 0.5	**58**	14.6	− 0.5
19	16.3	0.2	**39**	16.3	− 0.5	**59**	14.3	− 0.5
20	17	0.1	**40**	16	− 0.5	**60**	14	− 0.5

*CUSUM* Cumulative summation score

*The slope changed from positive to negative for the first time﻿

**Fig. 3:**
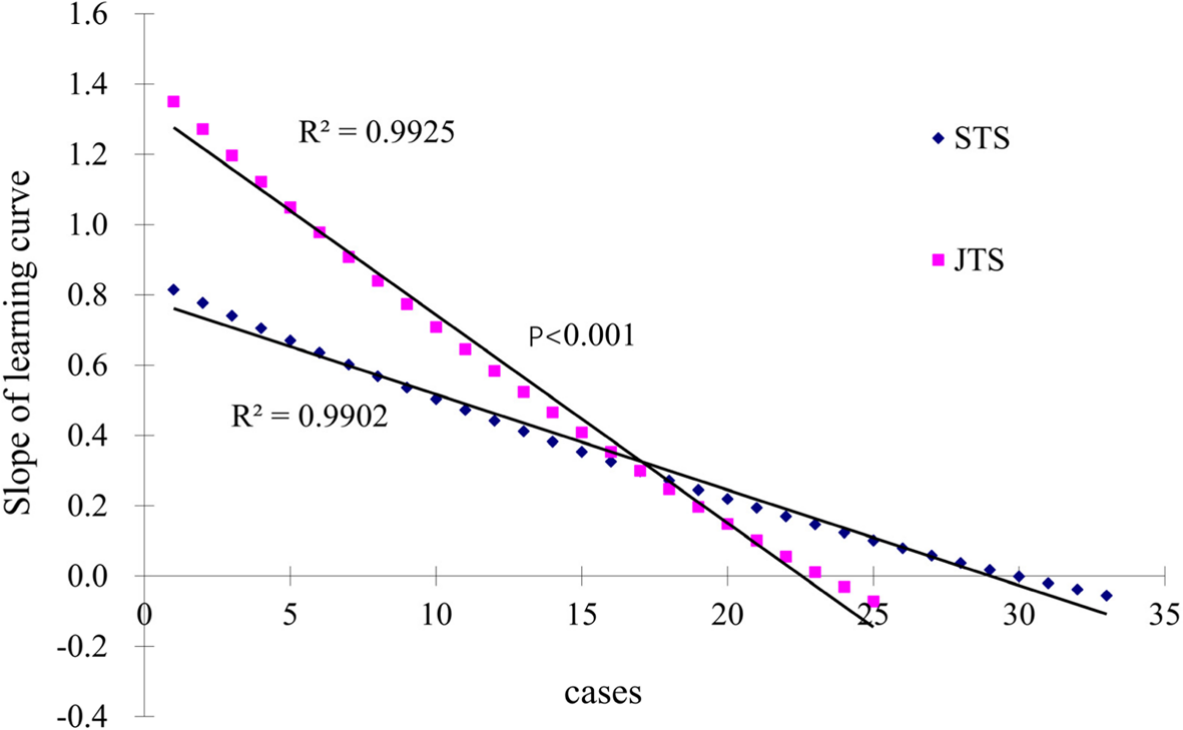
The slopes of learning curve for both groups in phase 1

The slopes in phase 1 were compared between both groups (Fig. 3), and the slope in the JTS group was greater than that in the STS group (*p* < 0.001). The comparisons of various parameters between both groups in phase 2 are presented in Table 4. Operation time, estimated blood loss, postoperative complications, and duration of postoperative hospital stay were similar between the groups, with no statistically significant difference (*p* > 0.05).

**Table 4:** The perioperative parameters between both groups in phase 2

Items	STS (*n* = 27)	JTS (*n* = 35)	*p*-value
Operation time (min)	216.5 ± 42.6	213.4 ± 52.4	0.15
Estimated blood loss (mL)	88.9 ± 48.9	92.9 ± 61.5	0.78
Lymph nodes harvested (*n*)	22.1 ± 6.8	22.0 ± 8.0	0.95
Chest tube duration (d)	5.0 ± 4.2	5.5 ± 3.1	0.90
Postoperative hospital stays (d)	7.0 ± 4.3	7.5 ± 3.8	0.10
Complications (*n*)	3	4	0.66
Atrial fibrillation	1	1	
Chylothorax	1	0	
Pulmonary embolism	0	1	
Pulmonary infection	1	2	

*CUSUM* cumulative summation score, *STS* senior thoracic surgeon, *JTS* junior thoracic surgeon

### Five-year survival rate

**Fig. 4:**
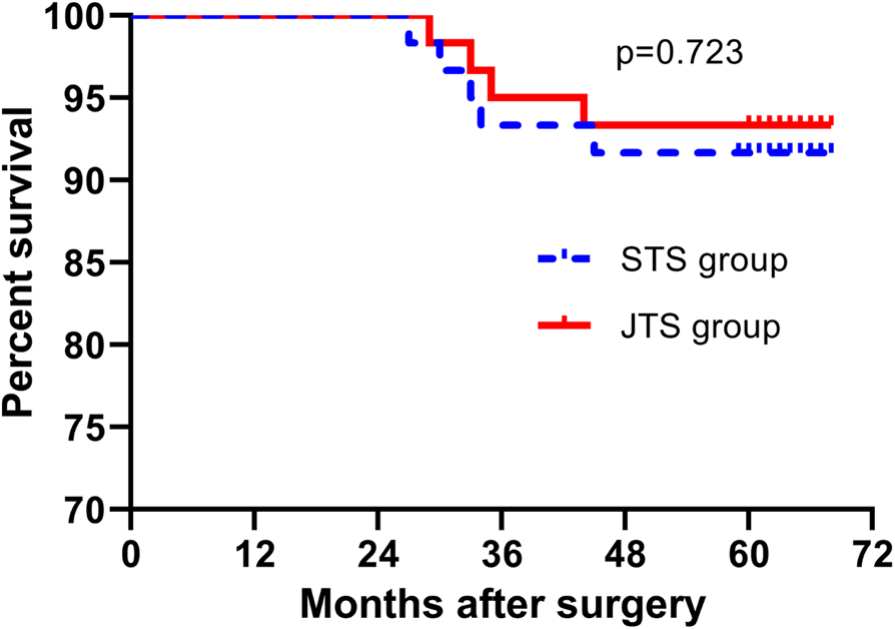
A 5-year overall survival between both groups in phase 2

In addition, the 5-year overall survival rate after overcoming the learning curve was 93.3% and 91.7% in the STS and JTS groups, respectively (Fig. 4), with no significant difference between the two groups (*p* = 0.72).

## Discussion

The learning curve refers to the gradual process of completing and mastering a certain skill through continuous learning. The general learning curve can be divided into two phases: fast-rise phase and platform phase. When the platform phase is reached, the surgeon’s technique is relatively skilled and stable, that is, it overcomes the learning curve. In recent years, the learning curve has been increasingly used to evaluate the acquisition of surgical skills and then guide the development of new surgical techniques [27, 28]. Learning curve studies mainly use the group-split method [15, 29]. However, there is no unified standard for group-split methods. The number of cases to overcome learning curves is relatively unclear, and several indicators of the learning curve evaluation are inconsistent between the groups.

CUSUM analysis has been used to analyze the learning curve of surgical procedures since the 1970s [30, 31]. CUSUM analysis transforms raw data into the running total of data deviations from their group mean, enabling investigators to visualize the data for trends that are not discernible using other approaches. It has been regarded as an aid for early assessment of surgical trainees and is already used in several fields [16].

Various factors can influence surgical procedures. However, most previous learning curve studies used operation time as the sole indicator [32]. The shortening of the operation time could demonstrate the proficiency of the surgical technique, and decrease in the estimated blood loss and duration of postoperative hospital stay could explain the improvement in the surgical technique [18]. Therefore, a single indicator, such as the operation time, may not result in an in-depth evaluation of the learning curve. In this study, those three indicators were combined and served as the evaluation criteria, and the learning curves were comprehensively drawn.

In the early stage, it was very difficult to carry out SPTL, due to the unskillful technique, inexperienced procedure, insufficient cooperation. Accordingly, in the initial cases, the operation time was long, the estimated blood loss was large, and the postoperative hospital stay was long as well. The CUSUM value gradually increased after accumulation. However, with improvements in surgical techniques, operative time, estimated blood loss, and duration of postoperative hospital stay all decreased. The CUSUM value gradually decreased in this period. As the slope of the learning curve transitioned from positive to negative, the exact cases reflected the mastery of the surgical procedure. In this study, 33 cases were required to overcome the SPTL learning curve in the STS group and 25 cases in the JTS group. The slopes of the learning curves in phase 1 were compared between both groups, and the degree of the slope in the JTS group was greater than that in the STS group, which meant that the learning efficiency was greater among the JTS. Comparisons of the perioperative parameters between both groups in phase 2 showed no statistically significant difference, which testified that the learning curve had been overcome. Although 8 fewer cases were required in the JTS group to overcome the learning curve, the average operation time was longer in the JTS group than in the STS group.

The reasons for better learning curves in younger surgeons are not completely defined. Senior surgeon may have a wealth of experience and a proven track record but may struggle to adapt to new techniques and technologies, due to their age, fixed mindset, and surgical mode, and minimally invasive surgery evolved from open surgery. On the other hand, junior surgeons may have more interest in innovative ideas and be more familiar with newer technologies. The basic skills are generally considered to be of great influence on learning curves; however, junior doctors can be easily trained to improve their experience with more recent methods, including video training, long-term advanced formation, self-determination training, simulator training, and webcast learning. Hence, an expert consensus about uniportal video-assisted thoracic surgery for lung cancer treatment reported that the experience of thoracotomy or multi-portal video-assisted thoracic surgery would not affect the learning curve [33].

In addition, conventional learning curve studies mainly focused on the evaluation of perioperative parameters. However, Berfield et al. suggested that the overall survival should be regarded as another indicator of the learning curve [18]. In accordance with their findings, we believe that oncology surgeons should not only perform a surgery but also pay attention to whether the surgery could bring oncological benefits. These differences might not be distinguished during the perioperative period; however, the postoperative overall survival and disease-free survival rates for the same tumor-staged operable patients could, to some extent, reflect these differences several years later. In this study, there was no difference in the 5-year overall survival rate between the two groups, which further confirmed that the surgeons successfully overcame the learning curve.

However, this study has some limitations including its single-center retrospective design and relatively small sample. Besides, bias for patient cohort selection inevitably remained. Regarding the learning curve for a new technology, we tend to start with some relatively simple cases, rather than complex cases. The failure of a newly developed surgical technique will lead to, on the one hand, grave damage to the interests of patients, and, on the other hand, it will also seriously hit the confidence of the surgeon. Moreover, the skill of the surgical procedure (especially in SPTL) depends on the whole surgical team members, which include the surgeon and the assistants. In our center, the assistants are made up of three residents, and they participate in all operations together. That is, although the assistants were not the same for every surgery, they were randomly involved in surgeries of both groups. To some extent, their effect on the whole study could be balanced or offset. Of course, controlling for several confounding factors would help decrease the selection bias. In the future, as an expanding number of scholars participate in this field of research, an increasingly objective and comprehensive exposition of this subject will be presented.

Generally, we conclude that the multidimensional CUSUM method is an effective tool for the objective evaluation of practical skills for surgeons during the learning phase of SPTL training. The data indicated that after a learning curve phase of 25–33 cases, thoracic surgeons could become increasingly skillful. Junior surgeons became competent in this new technology after about 25 cases, becoming more proficient in performing more complex surgeries. In summary, SPTL was found to have a shorter learning curve for JTSs.

## Data Availability

The datasets used and/or analyzed during the current study are available from the corresponding author on reasonable request.
